# Crystal structure of (1*R*,3a*R*,7a*R*)-1-{(*S*)-1-[(2*R*,5*S*)-5-(3-hy­droxy­pentan-3-yl)tetra­hydro­furan-2-yl]eth­yl}-7a-methyl-2,3,3a,4,5,6,7,7a-octa­hydro-1*H*-inden-4-one

**DOI:** 10.1107/S2056989016020648

**Published:** 2017-01-06

**Authors:** Andrea Martínez, Hugo Santalla, Fátima Garrido, Aliou Hamady Barry, Mohamed Gaye, Yagamare Fall Diop

**Affiliations:** a Dipartamento Química Orgínica, Facultade de Química, Universidade de Vigo, E-36310, Vigo, Spain; b Département de Chimie, Faculté des Sciences, Université de Nouakchott, Nouakchott, Mauritania; c Département de Chimie, Faculté des Sciences et Techniques, Université Cheikh Anta Diop, Dakar, Senegal

**Keywords:** crystal structure, calcitriol, vitamin D, hydrogen bonding

## Abstract

The title compound, C_21_H_36_O_3_, contains an oxolane ring, and six defined stereocentres and may serve as a useful synthon for the synthesis of calcitriol analogues. The configurations of the chiral C atoms of the side chain were unambiguously established in the refinement. In the crystal, C—H⋯O and extremely weak O—H⋯O hydrogen bonds arising from the sterically hindered alcohol group link the mol­ecules into a three-dimensional network.

## Abstract 2

The title compound contains an oxolane ring, and six defined stereocentres and may serve as a useful synthon for the synthesis of calcitriol analogues. The configurations of the chiral C atoms of the side chain were unambiguously established in the refinement.

## Chemical context   

The discovery of vitamin D3 (calcitriol) and its biological activity had a very important impact in the search for analogues of Vitamin D. In the structure of vitamin D, it is recognized that the side chain is the main site of metabolic degradation. Synthetic chemists have devoted considerable efforts to varying this chain in order to prepare analogues of vitamin D (Dai & Posner, 1994 ▸; Zhu *et al.*, 1995 ▸; Posner & Kahraman, 2003 ▸) and study the degradation metabolisms of these new mol­ecules. Our ongoing inter­est in the chemistry of heterocyclic compounds, and particularly in the synthesis of vitamin D analogues, has led us to develop several methods for the synthesis of these compounds (Fernández *et al.*, 2016 ▸; Gándara *et al.*, 2009 ▸). We have also looked at their biological activities which are reported in the literature (Maehr *et al.*, 2004 ▸). Recently, we reported the synthesis of a new vitamin D2 analogue and the evaluation of its biological activity on colon cancer (Gándara *et al.*, 2012 ▸). In a continuation of our work on the analogues of vitamin D, we synthesized two new mol­ecules of cacitriol from an oxolane ring and its side chains (Martínez *et al.*, 2013 ▸). In this study we present the structure of a new analog of calcitriol with six stereo centres.
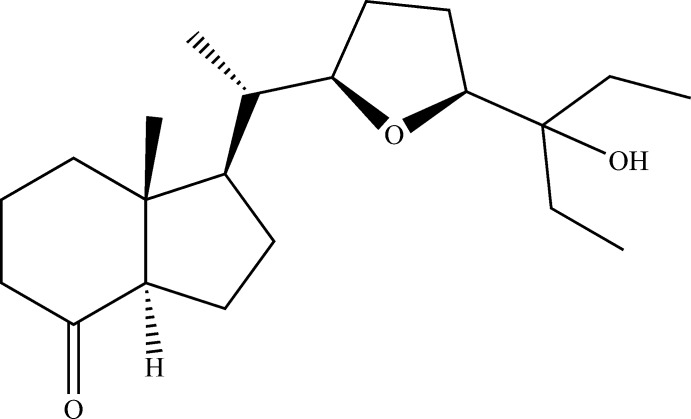


## Structural commentary   

**Figure 1:**
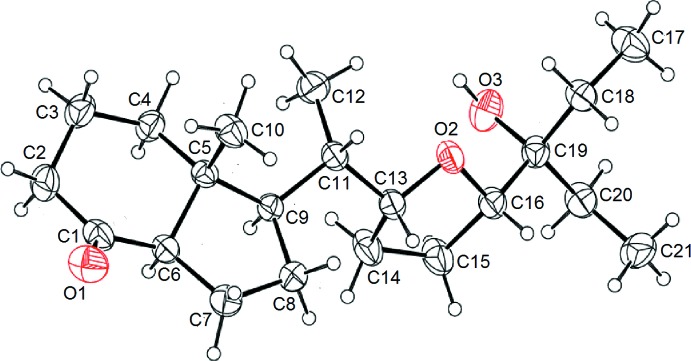
An *ORTEP* view of the title compound with displacement ellipsoids plotted at the 50% probability level.

The mol­ecular structure of the title compound is shown in Fig. 1 ▸: the compound crystallizes in the non-centrosymmetric space group *P*2_1_ and the absolute structure was unambiguously established. The mol­ecule contains a cyclo­pentane ring *trans*-fused to a cyclo­hexa­none ring. The lateral chain contains an oxolane ring. The cyclo­hexa­none ring adopts a chair conformation, the cyclo­pentane ring is an envelope (flap atom = C5) and the heterocyclic ring is twisted about C13—O2. The configurations of the stereogenic centres are C5(*R*), C6(*R*), C9(*R*), C11(*S*), C13(*R*) and C16(*S*). All bond distances and angles are within their expected ranges. The C*sp* ^3^—C*sp* ^2^ bonds involving C1 [1.499 (3) and 1.500 (3) Å) are naturally slightly shorter than the C*sp* ^3^—C*sp* ^3^ bonds [1.514 (3)–1.549 (5) Å]. The C1=O1 bond length [1.208 (3) Å] is typical of a C=O double bond, confirming oxidation of the starting alcohol.

## Supra­molecular features   

**Figure 2:**
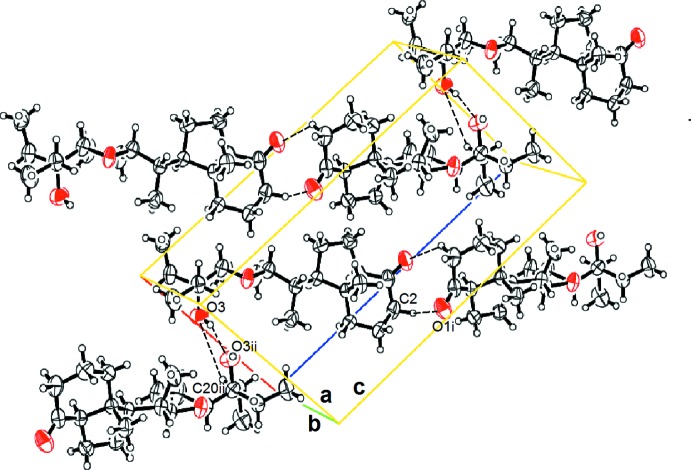
The packing of the title compound showing hydrogen bonds as dashed lines. [Symmetry codes: (i)-*x* + 2, *y* − 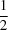, −*z* + 1, (ii)-*x* + 1, *y* + 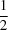, −*z* + 2.]

In the crystal, C2—H2*B*⋯O1=C hydrogen bonds (Table 1 ▸, Fig. 2 ▸) link the mol­ecules into *C*(4) chains, which propagate parallel to [101]. The chains are linked through very weak *C*(2) O3—H3*O*⋯O3 hydrogen bonds, giving rise to a three-dimensional supra­molecular architecture. The O—H⋯O hydrogen bond is very long, presumably due to steric hindrance of the –OH group.

## Database survey   

A survey of the Cambridge Structural Database (Version 5.38, last update Nov 2016; Groom *et al.*, 2016 ▸) for the bicyclic moiety fragment (1*S*,3a*R*,7a*R*)-1-ethyl-7a-methyl-octa­hydro­inden-4-one) of the title compound revealed just three matches, *viz*. EFEHEE (Pietraszek *et al.*, 2013 ▸), LESNEE (Rivadulla *et al.*, 2013 ▸) and ZEBZIP (Schwarz *et al.*, 1995 ▸). In each case, the shared C—C bond of the [4.3.0]-bicyclic moiety presents a *trans* configuration, as does the structure reported here.

## Synthesis and crystallization   

To a solution of diol **2** (0.18 mmol) in CH_2_Cl_2_ (5 ml), pyridinium dichromate (PDC) (0.37 mmol) was added, and the mixture stirred at room temperature for 12 h, then the solvent was evaporated and the residue was chromatographed on sílica gel using (10% EtOAc/hexa­ne) to afford ketone **1**. The title compound was recrystallized as colourless blocks using a solvent mixture of hexa­ne/ethyl ether (1:1).
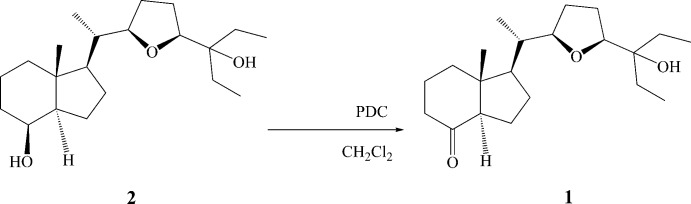


Compound **1**: white solid; m.p. 382–384 K. yield: 83%; *R*_f_: 0.54 (30% EtOAc/hexa­ne). [α]_20_^D^ = +31.39° (*c* 1.0, CDCl_3_). ^1^H NMR (CDCl_3_, δ): 3.87 (1H, *m*, H-5′), 3.72 (1H, *m*, H-2′), 2.44 (1H, *dd*, *J* = 11.2, 7.4 Hz), 2.49–1.8 (6H, *m*), 1.79–1.65 (4H, *m*), 1.65–1.28 (8H, *m*), 1.27 (3H, *d*, *J* = 9.7 Hz), 0.95 (3H, *d*, *J* = 6.7 Hz, CH_3_-21), 0.88 (6H,*q*, *J* = 7.6 Hz, CH_3_-Et), 0.67 (3H, *S*, CH_3_-18). ^13^C NMR (CDCl_3_, δ): 211.91 (C=O), 82.17 (CH-2′), 80.67 (CH-5′), 74.96 (C-3′′), 61.49 (CH-14), 54.50, 50.25 (CH-17, CH-13), 41.01 (CH_2_), 38.97 (CH_2_), 38.12 (CH-20), 28.65 (CH_2_), 26.93 (CH_2_), 26.23 (CH_2_), 24.98 (CH_2_), 24.52 (CH_2_), 24.04 (CH_2_), 19.21 (CH_2_), 12.70 (CH_3_-21), 12.55 (CH_3_-18), 8.02 (CH_3_-Et), 7.52 (CH_3_-Et). IR (NaCl, cm^−1^): 3532, 2964, 2939, 2881, 2347, 1714, 1460, 1381, 1246, 1136,1077, 958, 837. MS (ESI^+^) [*m*/*z*, (%)]: 359.25 [(*M* + Na)^+^, (54)]; 319.26 [(*M* − OH)^+^,(100)]; 301.25 (15). HRMS (ESI^+^): calculated for C_21_H_36_NaO_3_, 359.25567 g mol^−1^; found: 359.2556 g mol^−1^.

## Refinement   

Crystal data, data collection and structure refinement details are summarized in Table 2 ▸. The hy­droxy H atom was located from a difference Fourier map and relocated to an idealized (O—H = 0.82Å) location. The other H atoms (CH, CH_2_ and CH_3_ groups) were placed geometrically and refined as riding atoms with *U*_iso_(H) = 1.2*U*_eq_(C) (1.5 for CH_3_ groups).

## Supplementary Material

Crystal structure: contains datablock(s) I, shelx. DOI: 10.1107/S2056989016020648/hb7644sup1.cif

Structure factors: contains datablock(s) I. DOI: 10.1107/S2056989016020648/hb7644Isup2.hkl

Supporting information file. DOI: 10.1107/S2056989016020648/hb7644Isup3.cml

CCDC reference: 1522774

Additional supporting information:  crystallographic information; 3D view; checkCIF report

## Figures and Tables

**Table 1:** Hydrogen-bond geometry (Å, °)

*D*—H⋯*A*	*D*—H	H⋯*A*	*D*⋯*A*	*D*—H⋯*A*
O3—H3*O*⋯O3^i^	0.82	2.67	3.4495 (9)	161
C2—H2*B*⋯O1^ii^	0.97	2.57	3.273 (3)	130

Symmetry codes: (i) 
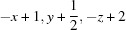
; (ii) 
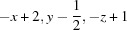
.

**Table 2:** Experimental details

Crystal data	
Chemical formula	C_21_H_36_O_3_
*M*_r_	336.50
Crystal system, space group	Monoclinic, *P*2_1_
Temperature (K)	296
*a*, *b*, *c* (Å)	9.4601 (3), 6.3779 (2), 16.7425 (4)
β (°)	104.196 (1)
*V* (Å^3^)	979.32 (5)
*Z*	2
Radiation type	Cu *K*α
μ (mm^−1^)	0.58
Crystal size (mm)	0.25 × 0.12 × 0.10

Data collection	
Diffractometer	Bruker *SMART* *APEX* CCD
Absorption correction	Multi-scan *SADABS* (Bruker, 2016 ▸)
*T*_min_, *T*_max_	0.662, 0.753
No. of measured, independent and observed [*I* > 2σ(*I*)] reflections	13069, 3679, 3594
*R*_int_	0.036
(sin θ/λ)_max_ (Å^−1^)	0.613

Refinement	
*R*[*F* ^2^ > 2σ(*F* ^2^)], *wR*(*F* ^2^), *S*	0.033, 0.097, 1.05
No. of reflections	3679
No. of parameters	222
No. of restraints	1
H-atom treatment	H atoms treated by a mixture of independent and constrained refinement
Δρ_max_, Δρ_min_ (e Å^−3^)	0.14, −0.14
Absolute structure	Flack *x* determined using 1566 quotients [(*I* ^+^)−(*I* ^−^)]/[(*I* ^+^)+(*I* ^−^)] (Parsons et al., 2013 ▸)
Absolute structure parameter	−0.07 (7)

Computer programs: *APEX3* and *SAINT* (Bruker, 2016 ▸), *SHELXT* (Sheldrick, 2015*a* ▸), *SHELXL-2014/7* (Sheldrick, 2015*b* ▸) and *ORTEP-3 for Windows* (Farrugia, 2012 ▸).

## supplementary crystallographic information

### Crystal data

**Table:**

C_21_H_36_O_3_	*F*(000) = 372
*M**_r_* = 336.50	*D*_x_ = 1.141 Mg m^−^^3^
Monoclinic, *P*2_1_	Cu *K*α radiation, λ = 1.54178 Å
*a* = 9.4601 (3) Å	Cell parameters from 9988 reflections
*b* = 6.3779 (2) Å	θ = 2.4–28.6°
*c* = 16.7425 (4) Å	µ = 0.58 mm^−^^1^
β = 104.196 (1)°	*T* = 296 K
*V* = 979.32 (5) Å^3^	Block, colourless
*Z* = 2	0.25 × 0.12 × 0.10 mm

### Data collection

**Table:**

Bruker SMART APEX CCD diffractometer	3679 independent reflections
Radiation source: fine-focus sealed tube	3594 reflections with *I* > 2σ(*I*)
Detector resolution: 8.3333 pixels mm^-1^	*R*_int_ = 0.036
φ and ω scans	θ_max_ = 71.0°, θ_min_ = 2.7°
Absorption correction: multi-scan *SADABS* (Bruker, 2016)	*h* = −11→11
*T*_min_ = 0.662, *T*_max_ = 0.753	*k* = −7→7
13069 measured reflections	*l* = −20→20

### Refinement

**Table:**

Refinement on *F*^2^	Hydrogen site location: inferred from neighbouring sites
Least-squares matrix: full	H atoms treated by a mixture of independent and constrained refinement
*R*[*F*^2^ > 2σ(*F*^2^)] = 0.033	*w* = 1/[σ^2^(*F*_o_^2^) + (0.0549*P*)^2^ + 0.0933*P*] where *P* = (*F*_o_^2^ + 2*F*_c_^2^)/3
*wR*(*F*^2^) = 0.097	(Δ/σ)_max_ < 0.001
*S* = 1.05	Δρ_max_ = 0.14 e Å^−^^3^
3679 reflections	Δρ_min_ = −0.14 e Å^−^^3^
222 parameters	Extinction correction: SHELXL-2014/7 (Sheldrick 2015b), Fc^*^=kFc[1+0.001xFc^2^λ^3^/sin(2θ)]^-1/4^
1 restraint	Extinction coefficient: 0.0034 (10)
Primary atom site location: structure-invariant direct methods	Absolute structure: Flack *x* determined using 1566 quotients [(*I*^+^)-(*I*^-^)]/[(*I*^+^)+(*I*^-^)] (Parsons et al., 2013)
Secondary atom site location: difference Fourier map	Absolute structure parameter: −0.07 (7)

### Special details

**Table:**

Geometry. All esds (except the esd in the dihedral angle between two l.s. planes) are estimated using the full covariance matrix. The cell esds are taken into account individually in the estimation of esds in distances, angles and torsion angles; correlations between esds in cell parameters are only used when they are defined by crystal symmetry. An approximate (isotropic) treatment of cell esds is used for estimating esds involving l.s. planes.

### Fractional atomic coordinates and isotropic or equivalent isotropic displacement parameters (Å^2^)

**Table:**

	*x*	*y*	*z*	*U*_iso_*/*U*_eq_	
O1	0.9190 (2)	0.6570 (3)	0.46585 (10)	0.0761 (5)	
O2	0.41676 (15)	0.5028 (2)	0.80627 (9)	0.0555 (4)	
O3	0.44001 (17)	0.3383 (4)	0.97018 (11)	0.0768 (5)	
H3O	0.480163	0.453143	0.975052	0.115*	
C1	0.9416 (2)	0.6116 (3)	0.53792 (13)	0.0552 (5)	
C2	1.0862 (2)	0.6426 (4)	0.59799 (15)	0.0651 (6)	
H2A	1.147857	0.727399	0.572289	0.078*	
H2B	1.133002	0.507331	0.611064	0.078*	
C3	1.0739 (2)	0.7482 (4)	0.67748 (15)	0.0630 (6)	
H3A	1.054571	0.896302	0.667028	0.076*	
H3B	1.166385	0.735483	0.717955	0.076*	
C4	0.9536 (2)	0.6548 (4)	0.71321 (12)	0.0523 (4)	
H4A	0.981399	0.514090	0.732883	0.063*	
H4B	0.943984	0.738532	0.759957	0.063*	
C5	0.80678 (19)	0.6465 (3)	0.65022 (10)	0.0412 (4)	
C6	0.82896 (19)	0.5162 (3)	0.57614 (11)	0.0456 (4)	
H6	0.868329	0.380487	0.598623	0.055*	
C7	0.6765 (2)	0.4733 (4)	0.52444 (13)	0.0626 (6)	
H7A	0.673148	0.343325	0.493967	0.075*	
H7B	0.642601	0.586840	0.485945	0.075*	
C8	0.5838 (2)	0.4576 (4)	0.58867 (12)	0.0562 (5)	
H8A	0.502692	0.554932	0.575205	0.067*	
H8B	0.545476	0.316752	0.589496	0.067*	
C9	0.68560 (18)	0.5124 (3)	0.67337 (10)	0.0422 (4)	
H9	0.731850	0.380868	0.696278	0.051*	
C10	0.7521 (3)	0.8691 (3)	0.62341 (14)	0.0589 (5)	
H10A	0.653632	0.862000	0.590462	0.088*	
H10B	0.813277	0.930699	0.591728	0.088*	
H10C	0.755209	0.953355	0.671323	0.088*	
C11	0.6037 (2)	0.5963 (3)	0.73561 (12)	0.0474 (4)	
H11	0.553105	0.724547	0.712223	0.057*	
C12	0.7015 (3)	0.6528 (5)	0.81908 (14)	0.0695 (6)	
H12A	0.766322	0.538155	0.839097	0.104*	
H12B	0.642665	0.680515	0.857150	0.104*	
H12C	0.757405	0.775343	0.813787	0.104*	
C13	0.4871 (2)	0.4371 (3)	0.74389 (13)	0.0500 (4)	
H13	0.413816	0.429521	0.691234	0.060*	
C14	0.5382 (3)	0.2155 (4)	0.77004 (18)	0.0672 (6)	
H14A	0.633369	0.217278	0.808473	0.081*	
H14B	0.543273	0.130859	0.722683	0.081*	
C15	0.4227 (3)	0.1314 (4)	0.81102 (19)	0.0726 (7)	
H15A	0.467379	0.066789	0.863599	0.087*	
H15B	0.361078	0.028941	0.776109	0.087*	
C16	0.3342 (2)	0.3267 (3)	0.82279 (13)	0.0532 (5)	
H16	0.240605	0.323844	0.781623	0.064*	
C17	0.0673 (3)	0.1360 (5)	0.86855 (17)	0.0792 (8)	
H17A	0.077247	0.115555	0.813379	0.119*	
H17B	0.009832	0.259036	0.870492	0.119*	
H17C	0.020167	0.016174	0.885197	0.119*	
C18	0.2170 (3)	0.1631 (4)	0.92637 (15)	0.0613 (5)	
H18A	0.272883	0.036389	0.924591	0.074*	
H18B	0.205358	0.177567	0.982067	0.074*	
C19	0.3051 (2)	0.3499 (3)	0.90802 (13)	0.0506 (4)	
C20	0.2324 (2)	0.5598 (3)	0.91465 (13)	0.0543 (5)	
H20A	0.298507	0.670669	0.907616	0.065*	
H20B	0.145850	0.570583	0.869632	0.065*	
C21	0.1888 (3)	0.5971 (5)	0.99509 (15)	0.0750 (7)	
H21A	0.153005	0.737676	0.995988	0.113*	
H21B	0.272144	0.577529	1.040541	0.113*	
H21C	0.113814	0.499588	0.999528	0.113*	

### Atomic displacement parameters (Å^2^)

**Table:**

	*U*^11^	*U*^22^	*U*^33^	*U*^12^	*U*^13^	*U*^23^
O1	0.1052 (13)	0.0728 (11)	0.0592 (9)	−0.0108 (10)	0.0371 (9)	0.0039 (8)
O2	0.0597 (8)	0.0400 (7)	0.0789 (9)	0.0002 (6)	0.0399 (7)	−0.0013 (6)
O3	0.0565 (8)	0.0872 (12)	0.0808 (11)	0.0013 (9)	0.0058 (8)	0.0121 (10)
C1	0.0704 (12)	0.0458 (10)	0.0583 (11)	0.0007 (9)	0.0326 (10)	−0.0009 (9)
C2	0.0582 (11)	0.0691 (14)	0.0773 (14)	−0.0098 (11)	0.0346 (11)	0.0008 (12)
C3	0.0544 (11)	0.0678 (14)	0.0690 (13)	−0.0168 (10)	0.0191 (10)	−0.0012 (10)
C4	0.0473 (9)	0.0599 (11)	0.0501 (9)	−0.0094 (9)	0.0129 (8)	−0.0010 (9)
C5	0.0460 (8)	0.0364 (9)	0.0433 (8)	0.0003 (7)	0.0150 (7)	0.0002 (7)
C6	0.0499 (9)	0.0429 (9)	0.0469 (9)	0.0016 (8)	0.0177 (7)	−0.0020 (8)
C7	0.0580 (11)	0.0794 (16)	0.0504 (10)	−0.0021 (11)	0.0133 (9)	−0.0143 (10)
C8	0.0446 (9)	0.0656 (13)	0.0573 (11)	−0.0028 (9)	0.0106 (8)	−0.0132 (10)
C9	0.0417 (8)	0.0392 (9)	0.0475 (9)	−0.0008 (7)	0.0142 (7)	−0.0020 (7)
C10	0.0729 (13)	0.0402 (10)	0.0700 (13)	0.0075 (9)	0.0299 (11)	0.0051 (9)
C11	0.0493 (9)	0.0417 (9)	0.0565 (10)	−0.0005 (7)	0.0228 (8)	−0.0038 (8)
C12	0.0716 (13)	0.0848 (17)	0.0594 (12)	−0.0226 (13)	0.0300 (10)	−0.0189 (12)
C13	0.0483 (9)	0.0452 (10)	0.0623 (11)	−0.0007 (8)	0.0244 (8)	−0.0052 (8)
C14	0.0746 (14)	0.0435 (11)	0.0989 (18)	0.0047 (10)	0.0503 (14)	0.0000 (11)
C15	0.0794 (15)	0.0432 (11)	0.1116 (19)	−0.0006 (11)	0.0550 (15)	−0.0024 (12)
C16	0.0538 (10)	0.0430 (10)	0.0695 (12)	−0.0031 (9)	0.0277 (9)	−0.0007 (9)
C17	0.0825 (16)	0.0829 (18)	0.0812 (15)	−0.0360 (14)	0.0372 (13)	−0.0116 (14)
C18	0.0705 (13)	0.0470 (11)	0.0751 (13)	−0.0012 (10)	0.0345 (11)	0.0064 (10)
C19	0.0470 (9)	0.0462 (10)	0.0603 (11)	0.0007 (8)	0.0165 (8)	0.0039 (8)
C20	0.0638 (11)	0.0476 (10)	0.0560 (11)	0.0007 (9)	0.0233 (9)	0.0001 (8)
C21	0.1001 (18)	0.0679 (15)	0.0673 (14)	0.0002 (13)	0.0401 (14)	−0.0036 (12)

### Geometric parameters (Å, º)

**Table:**

O1—C1	1.208 (3)	C10—H10C	0.9600
O2—C13	1.432 (2)	C11—C12	1.518 (3)
O2—C16	1.433 (2)	C11—C13	1.531 (3)
O3—C19	1.437 (2)	C11—H11	0.9800
O3—H3O	0.8200	C12—H12A	0.9600
C1—C2	1.499 (3)	C12—H12B	0.9600
C1—C6	1.500 (3)	C12—H12C	0.9600
C2—C3	1.521 (3)	C13—C14	1.523 (3)
C2—H2A	0.9700	C13—H13	0.9800
C2—H2B	0.9700	C14—C15	1.523 (3)
C3—C4	1.530 (3)	C14—H14A	0.9700
C3—H3A	0.9700	C14—H14B	0.9700
C3—H3B	0.9700	C15—C16	1.540 (3)
C4—C5	1.525 (2)	C15—H15A	0.9700
C4—H4A	0.9700	C15—H15B	0.9700
C4—H4B	0.9700	C16—C19	1.525 (3)
C5—C10	1.540 (3)	C16—H16	0.9800
C5—C6	1.549 (2)	C17—C18	1.517 (4)
C5—C9	1.553 (2)	C17—H17A	0.9600
C6—C7	1.514 (3)	C17—H17B	0.9600
C6—H6	0.9800	C17—H17C	0.9600
C7—C8	1.548 (3)	C18—C19	1.527 (3)
C7—H7A	0.9700	C18—H18A	0.9700
C7—H7B	0.9700	C18—H18B	0.9700
C8—C9	1.546 (3)	C19—C20	1.521 (3)
C8—H8A	0.9700	C20—C21	1.521 (3)
C8—H8B	0.9700	C20—H20A	0.9700
C9—C11	1.539 (2)	C20—H20B	0.9700
C9—H9	0.9800	C21—H21A	0.9600
C10—H10A	0.9600	C21—H21B	0.9600
C10—H10B	0.9600	C21—H21C	0.9600
			
C13—O2—C16	106.49 (15)	C13—C11—H11	107.3
C19—O3—H3O	109.5	C9—C11—H11	107.3
O1—C1—C2	123.2 (2)	C11—C12—H12A	109.5
O1—C1—C6	123.6 (2)	C11—C12—H12B	109.5
C2—C1—C6	113.21 (17)	H12A—C12—H12B	109.5
C1—C2—C3	113.09 (18)	C11—C12—H12C	109.5
C1—C2—H2A	109.0	H12A—C12—H12C	109.5
C3—C2—H2A	109.0	H12B—C12—H12C	109.5
C1—C2—H2B	109.0	O2—C13—C14	103.51 (17)
C3—C2—H2B	109.0	O2—C13—C11	110.25 (15)
H2A—C2—H2B	107.8	C14—C13—C11	117.17 (17)
C2—C3—C4	113.19 (18)	O2—C13—H13	108.5
C2—C3—H3A	108.9	C14—C13—H13	108.5
C4—C3—H3A	108.9	C11—C13—H13	108.5
C2—C3—H3B	108.9	C13—C14—C15	104.13 (17)
C4—C3—H3B	108.9	C13—C14—H14A	110.9
H3A—C3—H3B	107.8	C15—C14—H14A	110.9
C5—C4—C3	112.45 (16)	C13—C14—H14B	110.9
C5—C4—H4A	109.1	C15—C14—H14B	110.9
C3—C4—H4A	109.1	H14A—C14—H14B	108.9
C5—C4—H4B	109.1	C14—C15—C16	104.18 (18)
C3—C4—H4B	109.1	C14—C15—H15A	110.9
H4A—C4—H4B	107.8	C16—C15—H15A	110.9
C4—C5—C10	110.68 (17)	C14—C15—H15B	110.9
C4—C5—C6	107.05 (14)	C16—C15—H15B	110.9
C10—C5—C6	111.29 (15)	H15A—C15—H15B	108.9
C4—C5—C9	116.71 (14)	O2—C16—C19	109.67 (16)
C10—C5—C9	111.36 (15)	O2—C16—C15	105.70 (15)
C6—C5—C9	99.07 (14)	C19—C16—C15	115.24 (19)
C1—C6—C7	120.41 (17)	O2—C16—H16	108.7
C1—C6—C5	111.94 (16)	C19—C16—H16	108.7
C7—C6—C5	104.92 (15)	C15—C16—H16	108.7
C1—C6—H6	106.2	C18—C17—H17A	109.5
C7—C6—H6	106.2	C18—C17—H17B	109.5
C5—C6—H6	106.2	H17A—C17—H17B	109.5
C6—C7—C8	103.70 (16)	C18—C17—H17C	109.5
C6—C7—H7A	111.0	H17A—C17—H17C	109.5
C8—C7—H7A	111.0	H17B—C17—H17C	109.5
C6—C7—H7B	111.0	C17—C18—C19	115.5 (2)
C8—C7—H7B	111.0	C17—C18—H18A	108.4
H7A—C7—H7B	109.0	C19—C18—H18A	108.4
C9—C8—C7	106.92 (16)	C17—C18—H18B	108.4
C9—C8—H8A	110.3	C19—C18—H18B	108.4
C7—C8—H8A	110.3	H18A—C18—H18B	107.5
C9—C8—H8B	110.3	O3—C19—C20	109.23 (18)
C7—C8—H8B	110.3	O3—C19—C16	109.91 (16)
H8A—C8—H8B	108.6	C20—C19—C16	110.00 (16)
C11—C9—C8	113.34 (14)	O3—C19—C18	104.20 (17)
C11—C9—C5	120.18 (15)	C20—C19—C18	113.14 (16)
C8—C9—C5	103.14 (14)	C16—C19—C18	110.19 (17)
C11—C9—H9	106.4	C21—C20—C19	115.33 (19)
C8—C9—H9	106.4	C21—C20—H20A	108.4
C5—C9—H9	106.4	C19—C20—H20A	108.4
C5—C10—H10A	109.5	C21—C20—H20B	108.4
C5—C10—H10B	109.5	C19—C20—H20B	108.4
H10A—C10—H10B	109.5	H20A—C20—H20B	107.5
C5—C10—H10C	109.5	C20—C21—H21A	109.5
H10A—C10—H10C	109.5	C20—C21—H21B	109.5
H10B—C10—H10C	109.5	H21A—C21—H21B	109.5
C12—C11—C13	111.35 (17)	C20—C21—H21C	109.5
C12—C11—C9	114.32 (16)	H21A—C21—H21C	109.5
C13—C11—C9	108.95 (15)	H21B—C21—H21C	109.5
C12—C11—H11	107.3		

### Hydrogen-bond geometry (Å, º)

**Table:**

*D*—H···*A*	*D*—H	H···*A*	*D*···*A*	*D*—H···*A*
O3—H3*O*···O3^i^	0.82	2.67	3.4495 (9)	161
C2—H2*B*···O1^ii^	0.97	2.57	3.273 (3)	130

Symmetry codes: (i) −*x*+1, *y*+1/2, −*z*+2; (ii) −*x*+2, *y*−1/2, −*z*+1.
